# Photon-counting detector computed tomography for the diagnosis of coronary arteriovenous fistula: a new finding after repair of a coronary fistula case

**DOI:** 10.1093/ehjcr/ytaf376

**Published:** 2025-08-05

**Authors:** Jiawei Liu, Yonggao Zhang

**Affiliations:** Department of Radiology, The First Affiliated Hospital of Zhengzhou University, No. 1, East Jianshe Road, Zhengzhou 450052, Henan, China; Department of Radiology, The First Affiliated Hospital of Zhengzhou University, No. 1, East Jianshe Road, Zhengzhou 450052, Henan, China

A 54-year-old woman was admitted to the hospital two years ago with recurrent chest tightness, which worsened with activity. The initial diagnosis on admission was ventricular premature contractions. Detailed results of various investigations were as follows: electrocardiogram results showed ventricular premature contractions, cardiac ultrasound results showed an abnormal left intraventricular shunt (considering a possible right coronary artery–left ventricular fistula), and energy-integrating detector coronary computed tomography angiography (CCTA) image showed a right coronary artery–left ventricular fistula and an aneurysm adjacent to the fistula at the end of the right coronary artery (*Panels A–C*). The patient’s condition was assessed and the patient underwent a coronary fistula repair and was discharged with symptomatic relief after the procedure. Shortly thereafter, the patient was admitted to the hospital with reoccurring chest tightness and underwent ultra-high-resolution (UHR) CCTA on the photon-counting detector computed tomography (PCD-CT) clearly showed a newly developed coronary arteriovenous fistula (CAVF) located apically. The CAVF is located between the great cardiac vein, which travels in the left interatrial sulcus towards the confluence of the coronary sinus, and the left anterior descending artery (*Panels D–F*).

Coronary arteriovenous fistula is a rare condition defined as an abnormal termination of a coronary artery. The aetiology of CAVF is congenital or acquired, with medically originated CAVF most often resulting from cardiovascular surgery or percutaneous interventions. Coronary arteriovenous fistula is an abnormal conduit between an artery and a vein. Patients with CAVF may develop symptoms at birth or later, depending on the type of fistula and the presence of collateral circulation. Depending on the number, size, location, haemodynamic profile, and potential complications of the fistula, symptoms such as chest tightness and heart failure may occur. Whether the aetiology of newly developed CAVF is congenital has not been clarified, but the reason why coronary arteriovenous fistulae are shown on postoperative imaging images is considered to be possibly haemodynamically related. The UHR imaging provided by PCD-CT has the advantages of fast imaging speed and high image quality, which helps to diagnose this abnormality. Photon-counting detector CT imaging cannot only clearly show the fine structure of coronary arteries but also help to detect early lesions. This non-invasive modality also offers significant utility for postoperative follow-up and monitoring.

**Figure:**
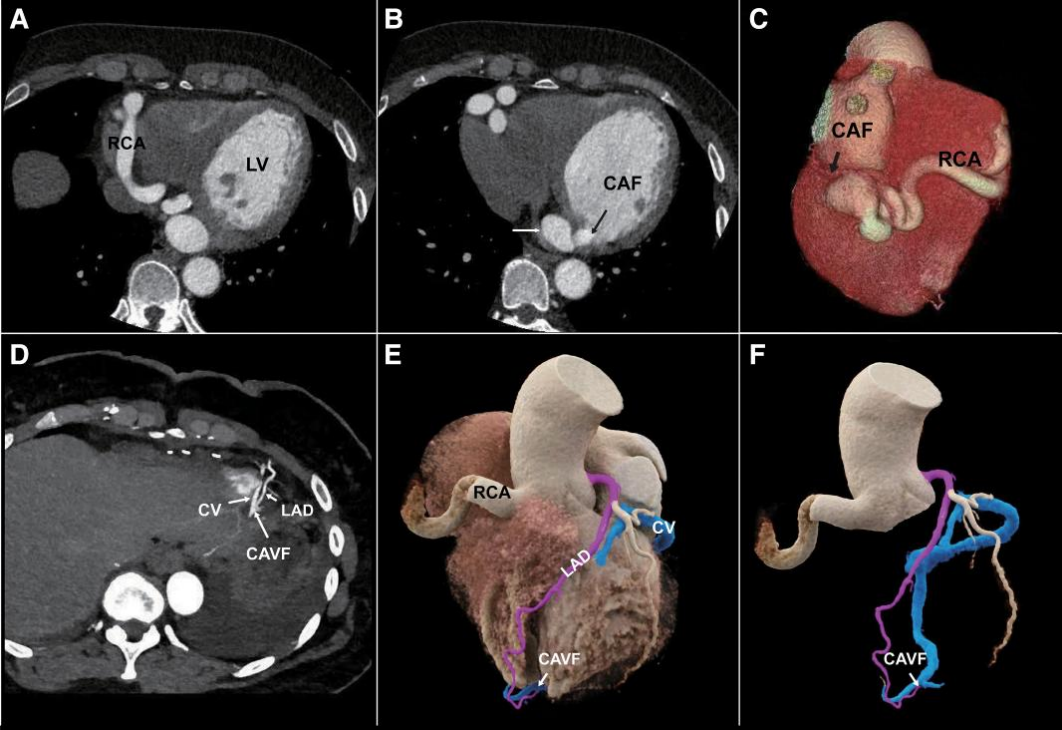


## Data Availability

The datasets used and/or analysed during the current study are available from the corresponding author on reasonable request.

## Notes

**Consent:** Appropriate informed consent was obtained from the patient for publication in accordance with the Committee on Publication Ethics (COPE) guidelines.

**Funding:** This study was supported by Henan Provincial Department of Science and Technology (231111313100).

